# Long noncoding RNA LINC00514 accelerates pancreatic cancer progression by acting as a ceRNA of miR-28-5p to upregulate Rap1b expression

**DOI:** 10.1186/s13046-020-01660-5

**Published:** 2020-08-08

**Authors:** Qing Han, Junhe Li, Jianping Xiong, Zhiwang Song

**Affiliations:** Department of Oncology, the First Affiliated Hospital of Nanchang University, 17 Yongwaizheng Street, Nanchang, Jiangxi 330006 People’s Republic of China

**Keywords:** LINC00514, Pancreatic cancer, Proliferation, Invasion, miR-28-5p, Rap1b

## Abstract

**Background:**

Pancreatic cancer (PC) is one of the most aggressive cancers and has an extremely poor prognosis worldwide. Long noncoding RNA (lncRNA) has been reported to be a potential prognostic biomarker in the initiation and prognosis of PC. Nevertheless, the biological functions and the detailed molecular mechanism of LINC00514 in PC remain unclear.

**Methods:**

We measured the expression level of LINC00514 in PC tissues and cell lines by quantitative real-time PCR. Gain- and loss-of-function experiments were performed to explore the bioeffects of LINC00514 on PC development both in vitro and in vivo. Subcellular fractionation, luciferase reporter assay, RNA immunoprecipitation assay, pull-down assay and western blotting were performed to investigate the oncogenic molecular mechanisms of LINC00514.

**Results:**

In this study, LINC00514 was shown to be upregulated in PC tissues and cell lines. Increased LINC00514 expression was significantly associated with the clinical progression and prognosis of PC patients. In addition, silencing LINC00514 inhibited PC cell proliferation, migration and invasion, while LINC00514 overexpression promoted these processes. Moreover, LINC00514 knockdown remarkably inhibited PC development and metastasis in vivo. Deeper investigations indicated that LINC00514 acted as a sponge for microRNA-28-5p (miR-28-5p) in PC and that Rap1b was a downstream target of miR-28-5p. Furthermore, the positive correlation of LINC00514 and Rap1b and the negative correlation between miR-28-5p and LINC00514 (or Rap1b) were revealed. Based on the rescue assays, Rap1b inhibition partially suppressed the oncogenic effect of LINC00514 overexpression on PC cell proliferation, migration and invasion.

**Conclusions:**

This study is the first to characterize the oncogenic function of the long noncoding RNA LINC00514 in pancreatic cancer progression by acting as a competing endogenous RNA (ceRNA) of miR-28-5p to upregulate Rap1b expression. Understanding this molecular mechanism might contribute to further discoveries of better diagnostic and therapeutic options for pancreatic cancer.

## Background

As an extremely aggressive cancer worldwide, pancreatic cancer (PC) has shown an increasing incidence rate in recent years [1]. Due to its high level of malignancy, PC has become the fourth leading cause of death from malignant tumors with poor prognosis, and the 5-year survival rate is less than 5% [2, 3]. Early diagnosis of PC has been a considerable challenge due to its complicated pathological process and intricate molecular mechanism. While some advances in imaging and clinical treatment have improved diagnosis and therapy [4], the outcome of PC patients remains unsatisfactory. Hence, a better understanding of the underlying molecular mechanism is essential for seeking a novel therapeutic target for PC.

Long noncoding RNA (lncRNA) is a ribonucleotide chain with a coding length of more than 200 nucleotides [5]. In the past, it was thought that since lncRNAs did not have the ability to encode proteins, they lacked biological functions. However, in recent years, scientists have found that lncRNAs execute their biological effects in epigenetics [6] at the histone modification [7], transcriptional and posttranscriptional levels [8]. Accumulated studies have elucidated the extraordinary significance of lncRNAs in the progression of a wide range of diseases, such as cardiovascular diseases [9], diabetes [10], neurodegenerative diseases [11] and human cancers. For instance, lncRNA SNHG1, which can be positively regulated by miR-21, activates the AKT pathway to promote sorafenib resistance in hepatocellular carcinoma cells [12]; lncRNA EPB41L4A-AS1 suppresses the Warburg effect and plays a significant role in metabolic reprogramming of cancer [13]; lncGata6 could maintain stemness of intestinal stem cells and promote tumorigenesis of colorectal cancer [14].

LINC00514 is a newly identified lncRNA, and very few reports about it are found in the literature. Research by Li et al [15] proved that LINC00514 could be an inhibitor of malignant behaviors of papillary thyroid cancer. In addition, another study has also shown a relationship between LINC00514 and neuroendocrine prostate cancer [16]. In our study, we explored the function and mechanism of LINC00514 in PC. We discovered that LINC00514 expression was increased in PC tissue and PC cell lines and that the upregulated expression of LINC00514 was associated with PC cell proliferation, migration and invasion in vitro and tumor growth and metastasis in vivo*.* Mechanistically, LINC00514 accelerated pancreatic cancer progression via the miR-28-5p/Rap1b axis. All the evidence above suggests that LINC00514 might act as a potential prognostic biomarker of PC occurrence and provide a novel target for PC therapy.

## Methods

### Clinical samples

PC tissue and adjacent normal tissue were collected from the First Affiliated Hospital of Nanchang University with the informed content of the enrolled patients in this research. Patients received neither chemotherapy nor radiotherapy before surgery. Our study was approved by the Human Research Ethics Committee of Nanchang University.

### Quantitative real-time PCR

RNA was extracted by TRIzol reagent (Invitrogen) from tissue samples and cells. Extracted RNA was later reverse transcribed into complementary DNA (cDNA) by PrimeScript RT Reagent (Takara, Japan). A SYBR Green Kit (Takara, Japan) was utilized to perform RT-PCR. GAPDH and β-actin were used as internal controls. Gene expression levels were calculated by the 2^−ΔΔCt^ method. The primer sequences are shown in Table 1.

**Table 1:** Primers involved in the study

Gene	Forward primer	Reverse primer
LINC00514	GAGGCAGGAGAATCGCTTGAACC	GAGGCAGGAGAATCGCTTGAACC
Rap1b	ACAGCAATGAGGGATTTATAC	TGACCTTGTTCCTTCCCTAC
MiR-28-5p	TGGTGTCGTGGGTCGA	CTCGCTTCGGCAGCACA
GAPDH	CGCTCTCTGCTCCTCCTGTTC	ATCCGTTGACTCCGACCTTCAC
U6	CTCGCTTCGGCAGCACA	AACGCTTVACGAATTTGCGT

*Abbreviation*: *LINC00514* long intergenic non-protein coding RNA 514, *GAPDH* glyceraldehyde 3-phosphate dehydrogenase

### Cell lines and cell culture

The normal pancreatic epithelial cell line (HPDE) and PC cell lines (BxPC-3, SW1990, PANC-1, AsPC-1, Capan-2, and MIAPaCa-2) were purchased from ATCC. Cells were cultured in Dulbecco’s modified Eagle’s medium (DMEM) containing 10% fetal bovine serum (FBS) at 37 °C with 5% CO_2_ in humidified air.

### Cell transfection

LINC00514 overexpression plasmid and shRNAs against LINC00514 and Rap1b were purchased from Gene Pharma (Shanghai, China) with scramble plasmid and shRNA used as negative controls. MiR-28-5p mimics and miR-28-5p inhibitors were acquired from Gene Pharma as well. All of the above reagents were transfected into cells via TF-3000 Transfection Reagent (Invitrogen) according to the manufacturer’s recommendations.

### Colony formation assay

Cells (1 × 10^3^ cells per well) were seeded in 6-well plates and then incubated for 10 days. After being washed with PBS three times, colonies were stained with hematoxylin and counted.

### Viability assay

To evaluate cell viability, a CCK-8 assay was carried out. Cells (1 × 10^5^ cells per well) were plated in 96-well plates for 0 h, 24 h, 48 h and 72 h. The cell growth rate was analyzed by Cell Counting Kit-8 (Solarbio, China) reagent according to the manufacturer’s instructions. The optical density value was measured by a microplate reader at 450 nm.

### Migration and invasion assays

A transwell chamber (Corning, Tewksbury, MA) was used to detect cell migration and invasion capacities. Cells (1 × 10^5^) were seeded on the upper chamber covered with Matrigel (Corning, Tewksbury, MA), while DMEM with 10% FBS was placed on the lower chamber. After 24 h of transfection, cells that passed from the upper chamber onto the lower chamber were fixed with methanol, stained with crystal violet and imaged under a light microscope.

### In vivo analysis

Five-week-old female nude mice were purchased from the National Laboratory Animal Center (Beijing, China) and maintained under specific pathogen-free conditions. Subsequently, the mice were randomly separated into two groups. Cells (1 × 10^6^) of the LINC00514 overexpression group and NC group were subcutaneously injected into the right axillary of nude mice. Tumor volume was measured every 4 days, and weight was measured at the end of the experiment.

To further evaluate the effects of LINC00514, we carried out pulmonary metastasis analysis. PC cells (1 × 10^6^) were injected into nude mice via the caudal vein. After 30 days, the mice were euthanized. The lungs of mice were removed to observe tumor metastasis. All experiments were approved by the Animal Research Ethics Committee of Nanchang University.

### Subcellular fractionation assay

The PARIS Kit (Life Technologies) was used to isolate nuclear and cytoplasmic RNAs according to the manufacturer’s protocol. Reverse transcription of extracted RNAs and RT-PCR were conducted as described before.

### Luciferase reporter assay

The online software StarBase3.0 http://starbase.sysu.edu.cn/was used to predict the binding sites of LINC00514 to miRNA-28-5p. Wild-type LINC00514 and mutant LINC00514 of the putative binding sites were cloned into a luciferase vector (Promega) and cotransfected with miR-28-5p mimics into PC cells via LF-3000 transfection reagent. After 48 h, cells were harvested for luciferase activity analysis.

### Pull-down assay

Wt-miR-28-5p and NC-miRNA were labeled with biotin and transfected into BxPC-3 and SW1990 cells. The cell lysates were incubated with streptavidin magnetic beads at 4 °C for 4 h. After that, the beads were rinsed with precooled lysis buffer and salt buffer. The pull-down RNAs were extracted to detect LINC00514 levels.

### RNA immunoprecipitation assay

The Magna RIP RNA-Binding Protein Immunoprecipitation Kit (Millipore, MA) was used to conduct the RIP assay according to the manufacturer’s protocol. The cells were lysed and incubated with Ago_2_ and IgG. Then, cell lysates were mixed with anti-Ago_2_ and anti-IgG in RIP buffer (Millipore). Precipitated RNAs were collected for RT-PCR analysis.

### Western blot

Protein was extracted from cells and transferred to polyvinylidene difluoride (PVDF) membranes after 10% sodium dodecyl sulfate polyacrylamide gel electrophoresis. After that, membranes were blocked with 5% nonfat milk and incubated overnight at 4 °C with primary antibodies. After rinsing the membranes three times with PBS, a secondary antibody labeled with horseradish peroxidase was used to incubate membranes for 2 h at room temperature. Antibodies against E-Cadherin, N-Cadherin, and Vimentin were all purchased from CST company, and β-actin and Rap1b antibodies were purchased from Abcam. The dilution ratio was determined according to the instructions.

### Statistics analysis

All data are presented as the mean ± standard deviation. All experiments were repeated at least three times. Student’s t test, ANOVA, Spearman’s rank correlation test and χ2 test were used for statistical analysis. A value of *p* < 0.05 was considered statistically significant.

## Results

### LINC00514 was upregulated in PC and predicted a poor prognosis

**Fig. 1:**
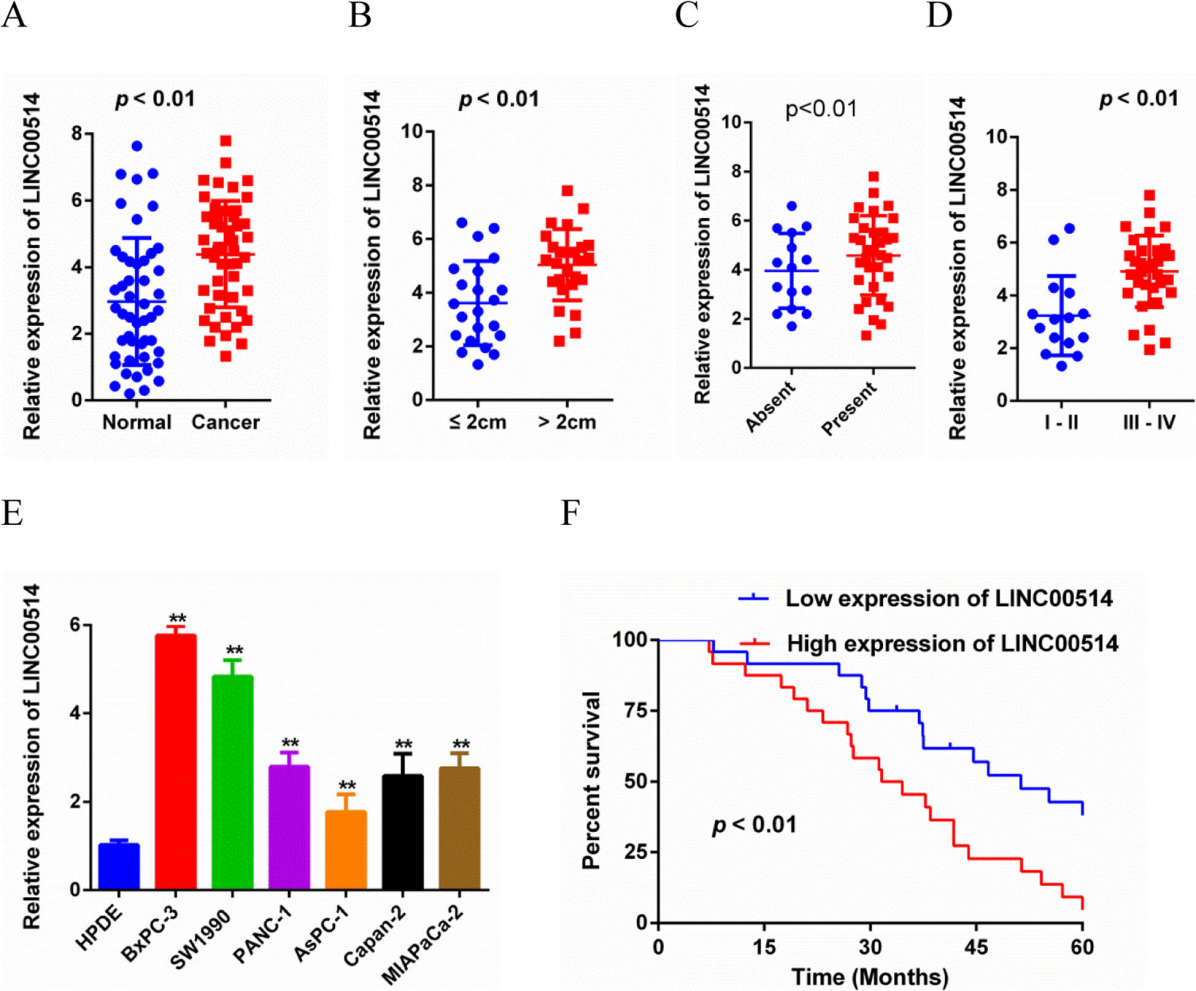
LINC00514 was up-regulated in PC and predicted a poor prognosis. (**a**) LINC00514 expression was detected in PC tissue and adjacent normal tissue by qRT-PCR. (**b**-**d**) Associations between LINC00514 expression and tumor size, Lymph node metastasis or clinical stage were detected by qRT-PCR. (**e**) qRT-PCR was applied to confirm the expression level of LINC00514 in PC cell lines and normal pancreatic epithelial cell line. (**f**) Kaplan-Meier analysis was used to assess the relation between LINC00514 expression level and overall survival in PC patients. *p<.05, **p<.01, **p<.001. All experiments were repeated at least for three times and mean ± SD was used to represent the final result. PC, pancreatic cancer; qRT-PCR, quantitative real-time polymerase chain reaction; SD, standard deviation

First, we analyzed the LINC00514 profile in PC. We found that LINC00514 was remarkably increased in PC tissues compared with the corresponding normal tissues (Fig. 1a). The upregulated expression of LINC00514 was significantly associated with the LINC00514 level and tumor size, lymph node metastasis and clinical stage (Fig. 1b-d), while no significant correlation was found between LINC00514 expression and age, gender or tumor differentiation (Table 2). Additionally, LINC00514 expression was increased in PC cell lines compared with the normal pancreatic epithelial cell line (Fig. 1e). Furthermore, Kaplan–Meier survival curves revealed that high LINC00514 expression was related to a lower overall survival rate compared with that of the low LINC00514 level group (Fig. 1f). Overall, LINC00514 was increased in PC and might be associated with clinical progression and a poor prognosis of PC patients.

**Table 2:** Correlation between LINC00514 expression level and clinical features

Characteristics	N^2^	LINC00514 expression		*P* value
		High	Low	
All	48	24	24	
Age (years)				0.628
< 60	22	12	10	
≥ 60	26	12	14	
Gender				0.472
Male	27	14	13	
Female	21	10	11	
Tumor size (cm)				0.019*
< 2	22	4	18	
≥ 2	26	20	6	
Differentiation				0.274
Poor	20	12	8	
Moderate/Well	28	12	16	
Lymph node metastasis				0.032*
Absent	15	5	10	
Present	33	19	14	
Clinical stage (AJCC)				0.003*
I-II	15	3	12	
III-IV	33	21	12	

*Abbreviation*: *LINC00514* long intergenic non-protein coding RNA 514

*p < 0.05 was considered statistically significant

### LINC00514 promoted cell proliferation, migration and invasion

**Fig. 2:**
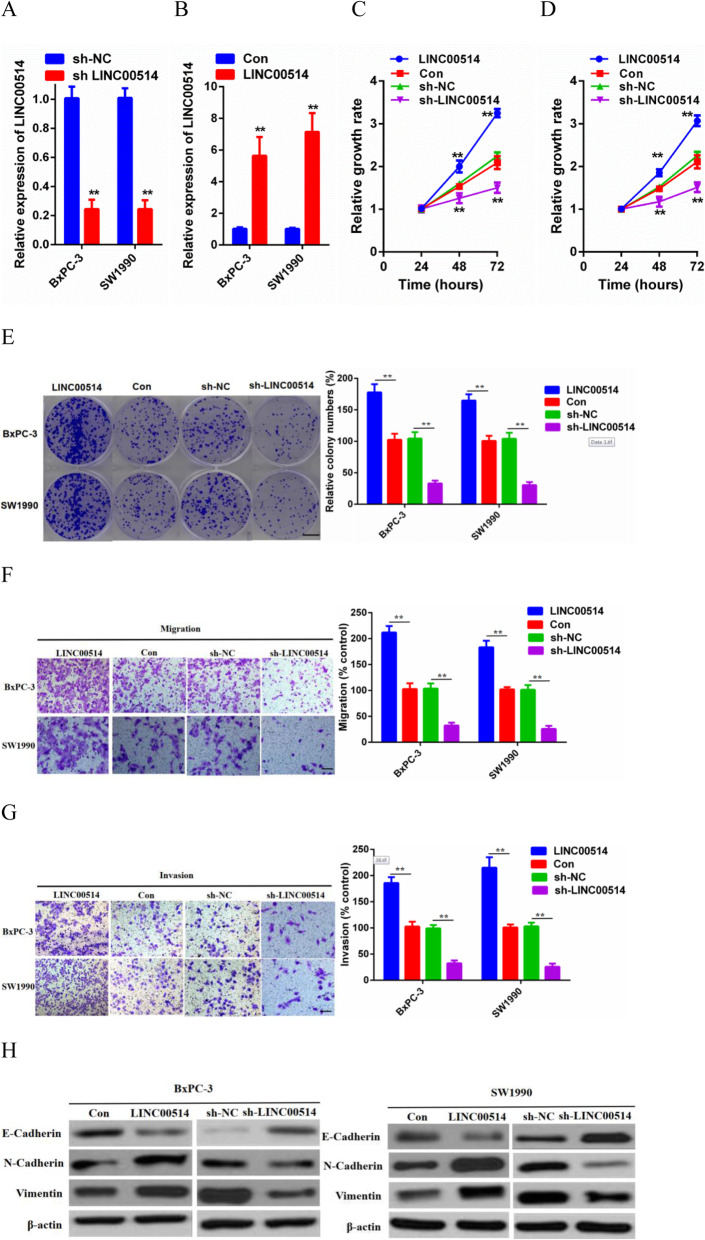
LINC00514 promoted PC cell proliferation, migration and invasion. (**a**-**b**) Transfection efficiency of LINC00514 overexpression plasmids and shRNA in BxPC-3 and SW1990 cells were evaluated by qRT-PCR. (**c**-**d**) CCK-8 assay was performed to detect cell (BxPC-3, SW1990) proliferation ability with LINC00514 overexpression and LINC00514 silencing. (**e**) Colony formation assay was carried out to further detect cell proliferation capacity. (**f**-**g**) Transwell migration and invasion assay were carried out to detect cell migration and invasion under LINC00514 overexpression and knockdown. (**h**) Western blot was conducted to evaluate the impact of LINC00514 on EMT progression. *p<.05, **p<.01, **p<.001. All experiments were repeated at least for three times and mean±SD was used to represent the final result. PC, pancreatic cancer; qRT-PCR, quantitative real-time polymerase chain reaction; SD, standard deviation; CCK-8, cell counting kit-8; EMT, epithelial-mesenchymal transition

To investigate whether LINC00514 is involved in cell proliferation, migration and invasion, we carried out gain- and loss-of-function assays. The LINC00514 overexpression plasmid and LINC00514 shRNA were stably transfected into BxPC-3 and SW1990 cells, with a scramble plasmid and shRNA used as negative controls (Fig. 2a-b). According to the results of CCK-8 and colony formation assays, LINC00514 overexpression significantly promoted cell (BxPC-3 and SW1990) proliferation capacity, while suppression of LINC00514 remarkably inhibited these processes (Fig. 2c-e). Moreover, transwell assays were utilized to prove that LINC00514 increased cell migration and invasion capabilities (Fig. 2f-g). For further confirmation, western blotting was performed to measure the expression of EMT markers in both BxPC-3 and SW1990 cells. As expected, E-cadherin was observed to be strikingly downregulated by LINC00514 overexpression, whereas N-cadherin and Vimentin were obviously upregulated, and the sh-LINC00514 group showed the opposite results (Fig. 2h). In summary, LINC00514 promoted PC cell proliferative, migratory and invasive capacities.

### LINC00514 knockdown inhibited tumor growth and pulmonary metastasis in vivo

**Fig. 3:**
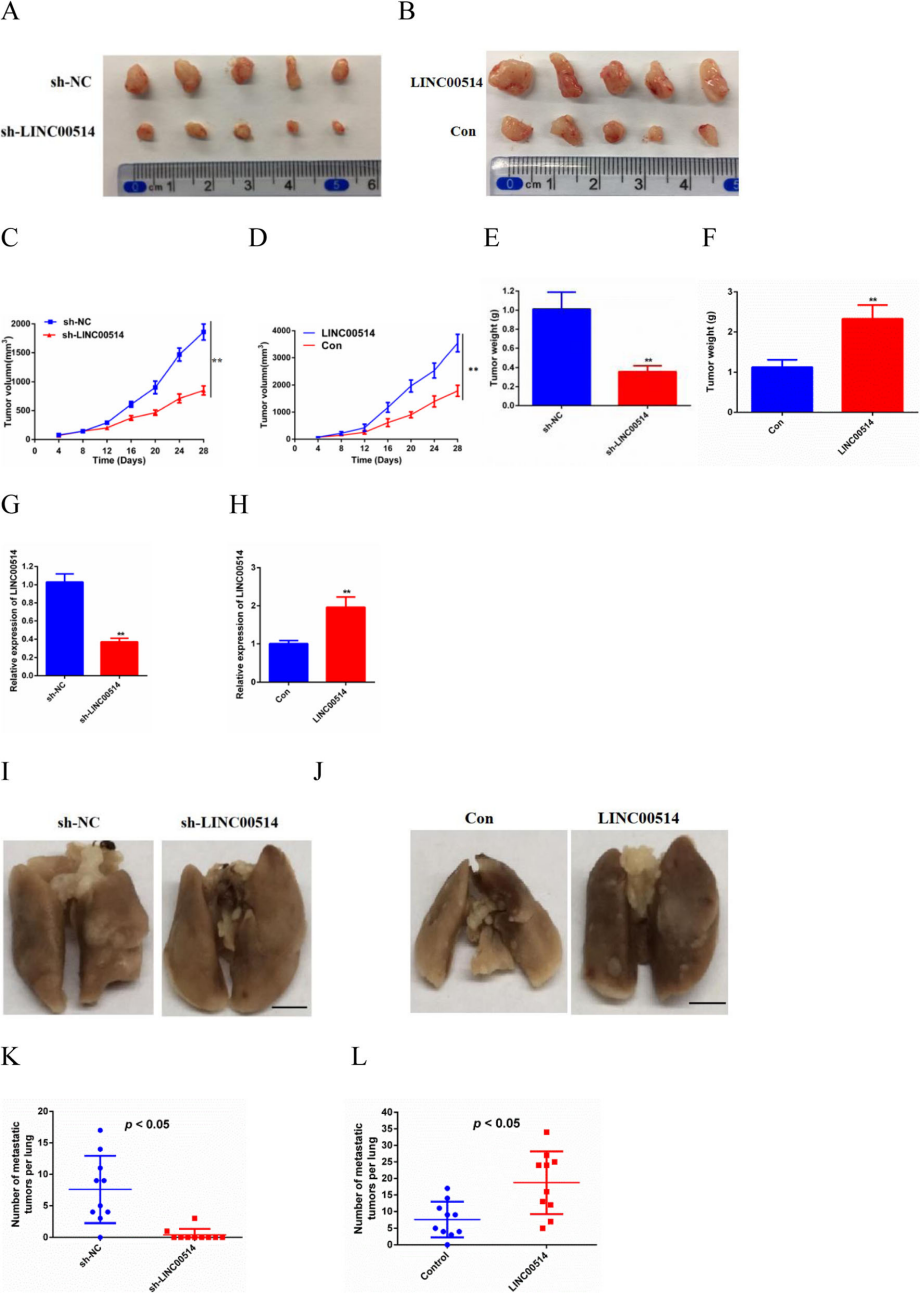
LINC00514 promoted tumor growth and pulmonary metastasis in vivo. (**a**-**b**) The images of subcutaneous tumors were obtained on Day 28. (**c**-**f**) The tumor volumes and weights of sh-LINC00514 group compared with NC group and LINC00514 overexpression group compared with empty group were quantified. Tumor volumes were analyzed by ANOVA. (**g**-**h**) qRT-PCR was used to assess the transfection efficiency. (**i**-**j**) The image of pulmonary metastasis was photographed at the endpoint. (**k**-**l**) Pulmonary metastasis of LINC00514 silencing and LINC00514 overexpression compared with their control groups were evaluated. *p<.05, **p<.01, **p<.001. The mean±SD was used to represent the final results of experiments repeated at least three times. PC, pancreatic cancer; qRT-PCR, quantitative real-time polymerase chain reaction; SD, standard deviation; NC, negative control; ANOVA, analysis of variance

To further identify the bioeffects of LINC00514 on tumor growth, we constructed a subcutaneous xenograft tumor model. BxPC-3 cells transfected with LINC00514 shRNA compared with NC shRNA or transfected with the LINC00514 overexpression plasmid and compared with the empty plasmid were subcutaneously injected into nude mice. The tumors were measured every 4 days after injection. After euthanizing the mice, we obtained images of the tumors (Fig. 3a-b). Compared with those of the NC group, the volume and weight of tumors in the LINC00514 shRNA group were significantly reduced, while the opposite results were observed in the LINC00514 overexpression group (Fig. 3c-f). QRT-PCR was used to assess the transfection efficiency (Fig. 3g-h). Then, we further investigated the role of LINC00514 in PC metastasis in vivo. Nude mice were injected with BxPC-3 cells transfected with LINC00514 shRNA compared with NC shRNA or the LINC00514 overexpression plasmid compared with the empty plasmid into the tail vein. Images of pulmonary metastasis were acquired at the endpoint (Fig. 3i-j). There was an obviously lower incidence of pulmonary metastasis and a smaller number of metastatic tumors per lung in the sh-LINC00514 group compared with the NC group, whereas the LINC00514 overexpression group showed the opposite results (Fig. 3k-l).

### LINC00514 acted as a sponge for miR-28-5p

**Fig. 4:**
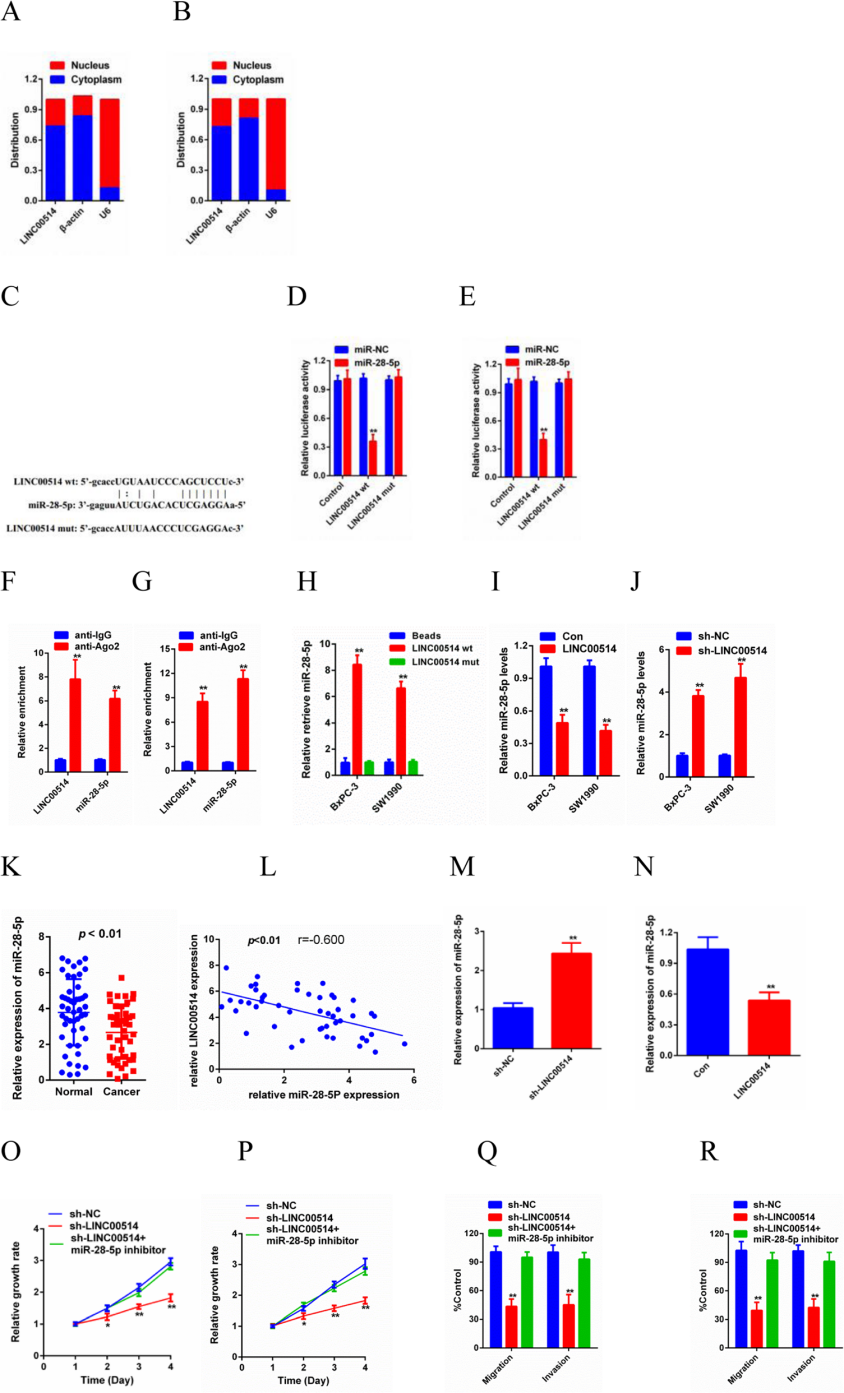
LINC00514 was a sponge for miR-28-5p. (**a**-**b**) Subcellular fractionation assay was used to determine the subcellular localization of LINC00514. **a**: BxPC-3 cells, **b**: SW1990 cells. (C) Sequence of WT-LINC00514, Mut-LINC00514 and miR-28-5p were conducted. (**d**-**g**) Luciferase reporter assay and RIP assay was performed to demonstrate that miR-28-5p was a downstream target of LINC00514. (**h**) Pull-down assay was conducted to detect the reaction between miR-28-5p and WT-LINC00514 or Mut-LINC00514. (**i**-**j**) Relative miR-18-5p expression level in BxPC-3 and SW1990 were determined by qRT-PCR. (K) The expression of miR-28-5p in PC tissue and normal tissue were detected by qRT-PCR. (L) Spearman’s rank correlation test was utilized to analyze the correlation between the levels of LINC00514 and miR-28-5p. (M-N) The miR-28-5p expression levels under LINC00514 silencing and LINC00514 overexpression were evaluated in vivo. (**o**-**p**) CCK-8 assay was performed to detect proliferation of cells transfected with LINC00514 shRNA and cells co-transfected with LINC00514 and miR-28-5p inhibitor. (**q**-**r**) Transwell migration and invasion assay were carried out to detect cell migration and invasion abilities. *p<.05, **p<.01, **p<.001. All experiments were repeated at least for three times and mean±SD was used to represent the final result. PC, pancreatic cancer; qRT-PCR, quantitative real-time polymerase chain reaction; RIP, RNA immunoprecipitation; SD, standard deviation; WT-lINC00514, wild type LINC00514; Mut-LINC00514, mutant LINC00514; CCK-8, cell counting kit‐8

To explore the underlying molecular mechanism of the oncogenic effects of LINC00514 on PC, we determined the subcellular localization of LINC00514. The results showed that LINC00514 was mostly distributed in the cytoplasm (Fig. 4a-b), which suggested that LINC00514 might exert its biological function by sponging miRNA. StarBase3.0 was utilized to identify a candidate microRNA (miR-28-5p) and predict the potential downstream targets of LINC00514 (Fig. 4c). The luciferase reporter assay results confirmed that the luciferase activity of WT-LINC00514 was clearly decreased by miR-28-5p mimics, while the luciferase activity of Mut-LINC00514 did not change significantly (Fig. 4d-e). In addition, the RIP assay further revealed that LINC00514 and miR-28-5p were enriched in beads conjugated to Ago_2_ compared with the IgG group (Fig. 4f-g). Furthermore, overexpression of WT-LINC00514, but not Mut-LINC00514, decreased miR-28-5p expression in BxPC-3 and SW1990 cells (Fig. 4h). Additionally, overexpressing LINC00514 dramatically decreased miR-28-5p levels in both BxPC-3 and SW1990 cells, while silencing LINC00514 increased miR-28-5p levels, with NC shRNA used as an internal reference (Fig. 4i-j). Then, we further detected miR-28-5p expression in tumor tissue. The results revealed a lower level of miR-28-5p in PC tissue than in normal tissue and a negative correlation between LINC00514 expression and miR-28-5p levels (Fig. 4k-l). To obtain more evidence, in vivo experiments were performed. We detected the expression level of miR-28-5p in the tumors we collected before, and the results showed that miR-28-5p expression was higher in the cell lines with LINC00514 knockdown, while miR-28-5p was lower in the cells with LINC00514 overexpression (Fig. 4m-n). LINC00514 promoted cell proliferation, migration and invasion at least partially by sponging miR-28-5p (Fig. 4o-r). In summary, LINC00514 accelerates PC progression by sponging miR-28-5p.

### Rap1b was a downstream target of miR-28-5p in PC

**Fig. 5:**
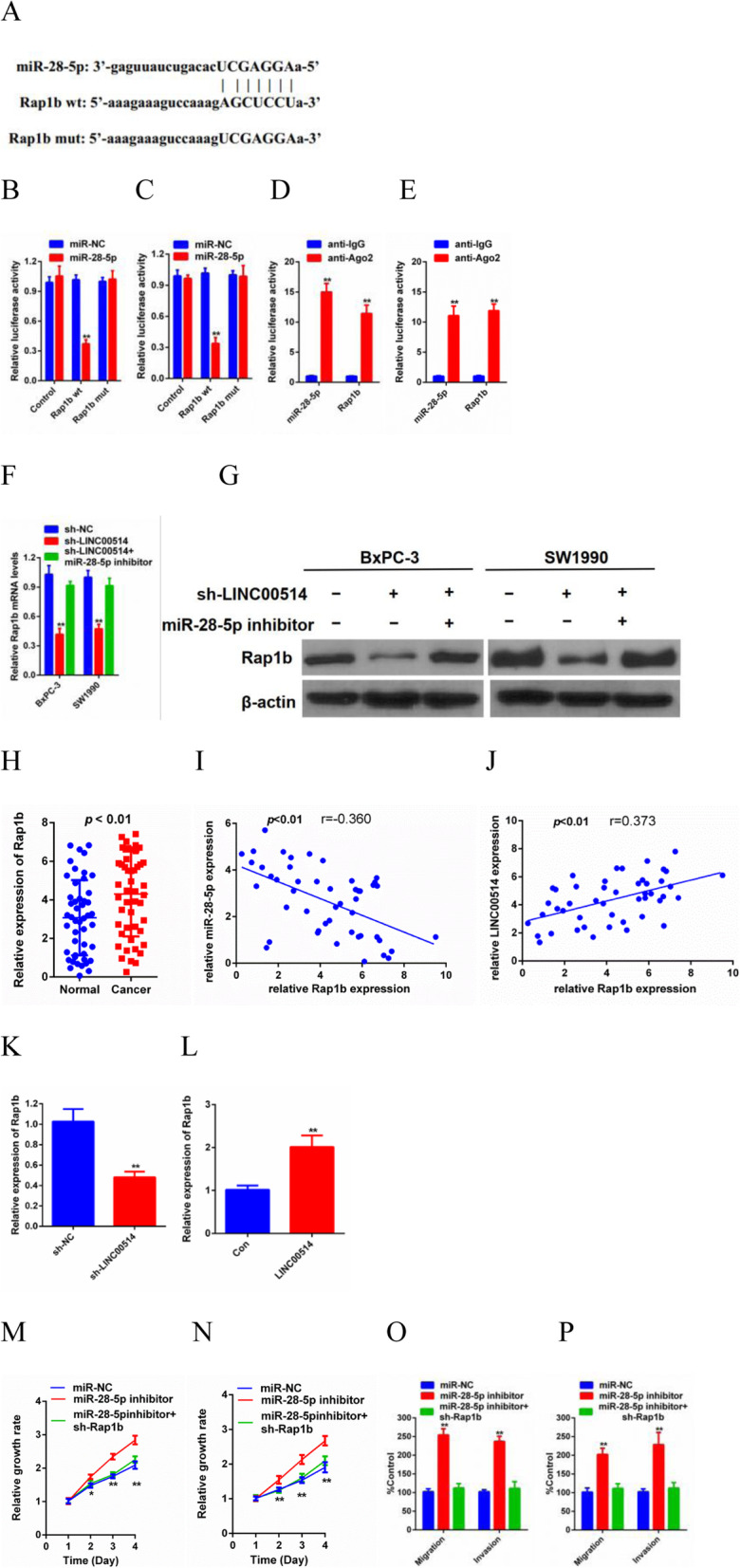
Rap1b was a downstream target of miR-28-5p in PC. (**a**) The sequence of WT-Rap1b, Mut-Rab1b and miR-28-5p were conducted. (**e**-**f**) Luciferase reporter assay and RIP assay were performed to determine the association between miR-28-5p and Rap1b. (**f**-**g**) QRT-PCR and western blot were used to detect Rap1b expression in cells of LINC00514 silencing and cells of co-transferring miR-28-5p and LINC00514 shRNA at transcription and translation level. (**h**) Relative Rap1b expression in tumor tissue and normal tissue were detected by qRT-PCR. (**i**-**g**) The correlation between Rap1b and LINC00514 as well as the correlation between Rap1b and miR-28-5p were analyzed by Spearman’s rank correlation test. (**k**-**l**) The Rab1b expression levels under LINC00514 silencing and LINC00514 overexpression were evaluated in vivo. (**m**-**n**) CCK-8 assay was performed to detect proliferation of cells transfected with miR-28-5p inhibitor and cells co-transfected with miR-28-5p inhibitor and Rap1b shRNA. (O-P) Transwell migration and invasion assay were carried out to detect cell migration and invasion abilities. *p<.05, **p<.01, **p<.001. All experiments were repeated at least for three times and mean±SD was used to represent the final result. PC, pancreatic cancer; qRT-PCR, quantitative real-time polymerase chain reaction; RIP, RNA immunoprecipitation; SD, standard deviation

The posttranscriptional function of miRNAs is usually to inhibit protein synthesis by base pairing with the 3′-untranslated region [17]. Next, to ascertain the detailed regulatory mechanism of LINC00514 in PC, we searched StarBase3.0 and observed that Rap1b was predicted to be a downstream target of miR-28-5p (Fig. 5a). Mut-Rap1b or WT-Rap1b and miR-28-5p or NC-miRNA were transfected into BxPC-3 and SW1990 cells. A luciferase reporter assay and RIP assay were used to confirm the hypothesis that Rap1b is a direct target of miR-28-5p (Fig. 5b-e). Then, we found that Rap1b expression was downregulated by LINC00514 silencing, while cotransfecting miR-28-5p and sh-LINC00514 inhibited the effect of LINC00514 knockdown on Rap1b at both the transcriptional and translational levels (Fig. 5f-g).

Then, we detected Rap1b levels in tumor tissue. Rap1b was obviously increased in PC tissue compared with adjacent normal tissue, and there was a positive relationship between Rap1b expression and LINC00514 levels, while a negative correlation was observed between Rap1b expression and miR-28-5p levels (Fig. 5h-j). In addition, we also detected the expression of Rap1b in vivo*,* and the transfection efficiency was examined previously. As expected, the expression of Rap1b was clearly decreased in BxPC-3 cells by LINC00514 knockdown, while increased expression was observed in LINC00514-overexpressing cells (Fig. 5k-l). Thus far, we have proven that Rap1b is a direct target of miR-28-5p. Thereafter, functional experiments were carried out to investigate the bioeffects of Rap1b. The results demonstrated that Rap1b silencing remarkably suppressed the promoting effects of the miR-28-5p inhibitor on cell proliferation, migration and invasion capacities (Fig. 5m-p).

### Rap1b inhibition inhibited the tumorigenesis effects of LINC00514

**Fig. 6:**
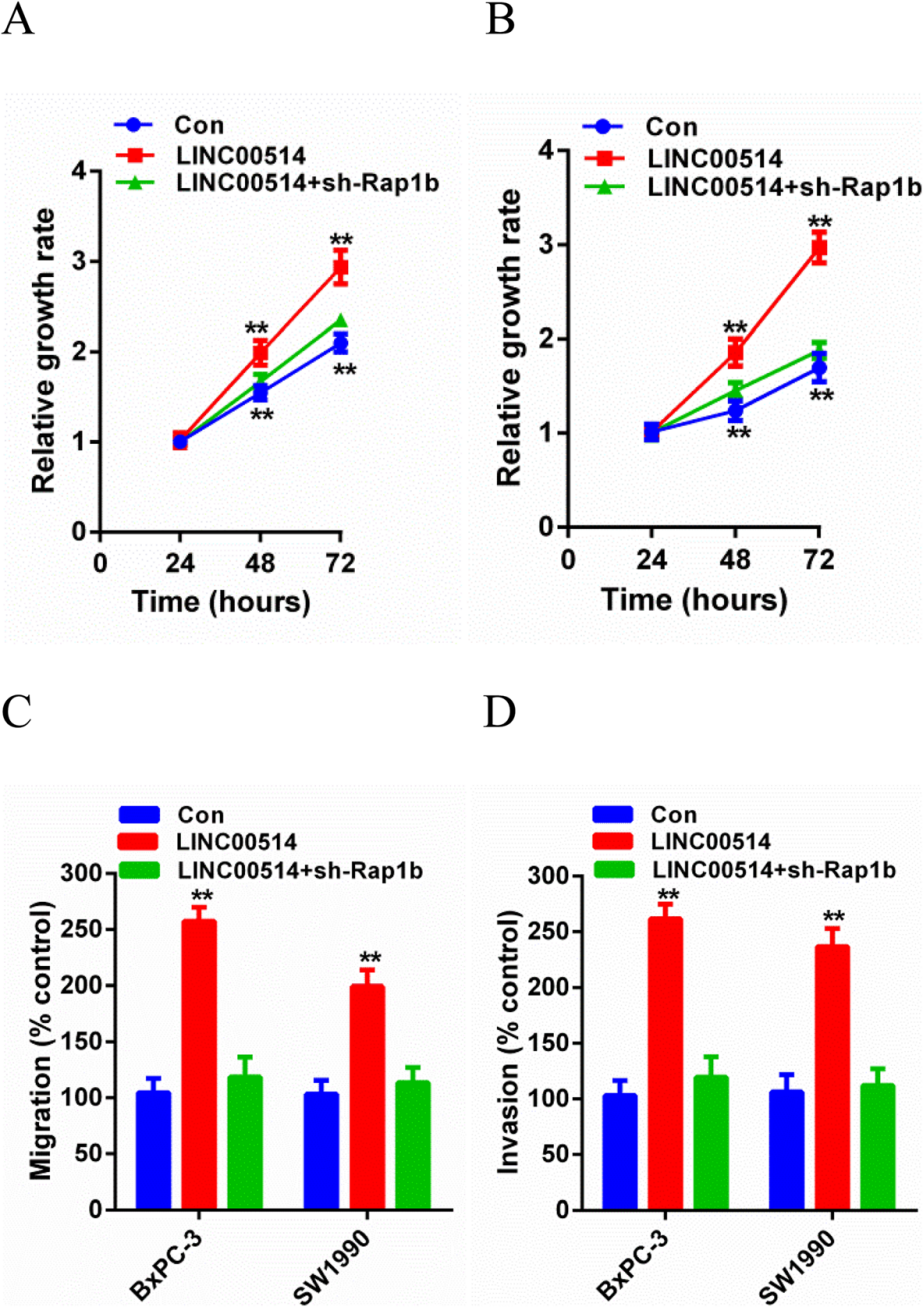
Rap1b inhibition restrained the tumorigenesis effects of LINC00514. (**a**-**b**) CCK-8 assay was performed to detect proliferation of cells transfected with LINC00514 overexpression plasmids and cells co-transfected with LINC00514 overexpression plasmids and Rap1b shRNA. (**c**-**d**) Transwell migration and invasion assay were carried out to detect cell migration and invasion abilities. *p<.05, **p<.01, **p<.001. All experiments were repeated at least for three times and mean±SD was used to represent the final result. PC, pancreatic cancer; qRT-PCR, quantitative real-time polymerase chain reaction; SD, standard deviation; CCK-8, cell counting kit-8

Finally, we explored the role of LINC00514 mediated by Rap1b in promoting tumor growth. We knocked down Rap1b in BxPC-3 and SW1990 cells to determine whether Rap1b inhibition can reverse the oncogenic effects of LINC00514. Based on the rescue assays, Rap1b inhibition partially inhibited the effect of LINC00514 overexpression on cell proliferation, migration and invasion (Fig. 6a-d). In conclusion, these results collectively demonstrated that LINC00514 acted as a key tumor promotor of PC by competitively binding to miR-28-5p and then upregulating the expression of Rap1b.

## Discussion

In recent years, PC has received increasing attention due to its high incidence and extremely poor prognosis [18]. Accumulated studies have shown that lncRNAs play an important role in the initiation and development of cancers, including PC [19]. For instance, LINC00346 accelerated PC progression and gemcitabine resistance partially through the miR-188-3p/BRD4 axis [20]; lncRNA GLS-AS mediated the feedback loop of Myc and GLS and provided a potential therapeutic strategy for metabolic reprogramming in PC [21]; AFAP1-AS1 was shown to exert inhibitory effects on the stemness of PC cells and ultimately PC tumorigenicity in vivo via the miR-384/ACVR1 axis [22]. LINC00514 has been previously reported in papillary thyroid cancer [15] and neuroendocrine prostate cancer [16], but there are no reports in PC. Our study revealed that LINC00514 expression was markedly elevated in PC tissues and PC cell lines and that increased expression of LINC00514 was associated with the progression and prognosis of PC patients, which indicated that LINC00514 might be involved in PC progression. In addition, it was determined that LINC00514 facilitated PC cell proliferation, migration and invasion in vitro and tumor growth and metastasis in vivo. The underlying molecules, however, have not yet been revealed.

In recent years, increasing evidence has proven the hypothesis that lncRNAs exert their biological impact by acting as competitive endogenous RNAs (ceRNAs) to affect the development of cancers [23]. There has been considerable progress in the study of ceRNAs in PC. For example, lncRNA-PVT1 promotes PC cell proliferation and migration by sponging miR-448 [24]; cucurbitacin B inhibits PC cell proliferation both in vitro and in vivo through lncRNA-AFAP1-AS1 binding with miR-146b-5p [25]; and PXN-AS1 acts as a ceRNA of miR-3064, which upregulates PIP4K2B expression and suppresses the progression of pancreatic cancer [26]. In our research, subcellular fractionation assays indicated that LINC00514 was mostly located in the cytoplasm, which provided a basis for LINC00514 to act as a ceRNA in the initiation and progression of PC. Then, the online software StarBase3.0 was utilized to predict the possible downstream target (miR-28-5p) for LINC00514. Luciferase reporter assay, RIP assay and pull-down assay were used to confirm the interaction between LINC00514 and miR-28-5p. Overexpression of LINC00514 suppressed miR-28-5p, while LINC00514 silencing upregulated miR-28-5p expression. Further investigation was carried out to demonstrate the migration and invasion promoting effect of miR-28-5p in the initiation and development of PC, which suggested a tumor-promoting effect of LINC00514 that was dependent on miR-28-5p.

According to the ceRNA hypothesis, mRNA expression is upregulated due to lncRNA competitively binding to miRNA. Rap1b was first reported in the study of Chajut et al. [27] and was found to be related to various cancers, such as thyroid cancer [28], breast cancer [29], gastric cancer [30], and colorectal cancer [31]. However, there is only a limited number of reports about Rap1b in PC [32]. In our current study, Rap1b was predicted to be a direct target of miR-28-5p by StarBase3.0. Luciferase reporter assay and RIP assay confirmed the direct binding of Rap1b with miR-28-5p. Rap1b, acting as a cancer-promoting gene, had a positive correlation with LINC00514, while there was a negative relationship between Rap1b and miR-28-5p. Moreover, silencing Rap1b partially abolished the tumorigenic effects of LINC00514 based on the rescue assay.

In conclusion, our study provides evidence that LINC00514 promotes PC development by sponging miR-28-5p and increasing Rap1b expression. This highlights the LINC00514/miR-28-5p/Rap1b axis as a novel diagnostic and therapeutic strategy for PC patients.

## Conclusions

Our results highlighted the significant role of the LINC00514/miR-28-5p/Rap1b axis in PC progression, suggesting that LINC00514 may serve as a potential biomarker and therapeutic target in PC.

## Data Availability

All the data and materials supporting the conclusions were included in the main paper.

## Abbreviations

PC: Pancreatic cancer
LncRNA: Long noncoding RNA
LINC00514: Long noncoding RNA00514
MiR-28-5p: MicroRNA-28-5p
CeRNA: Competing endogenous RNA
EMT: Epithelial-mesenchymal transition
QRT-PCR: Quantitative real-time PCR
RIP: RNA immunoprecipitation
